# HOW DO UNMET NEEDS VARY BETWEEN PEOPLE WITH AND WITHOUT DEMENTIA RECEIVING HOME AND COMMUNITY-BASED SERVICES

**DOI:** 10.1093/geroni/igae098.1942

**Published:** 2024-12-31

**Authors:** Eric Jutkowitz, Jack Wolf, Romil Parikh, Benjamin Langworthy, Tetyana Shippee

**Affiliations:** Brown University, Brown University, Rhode Island, United States; University of Minnesota, Minneapolis, Minnesota, United States; University of Minnesota, Minneapolis, Minnesota, United States; University of Minnesota, Minneapolis, Minnesota, United States; University of Minnesota School Of Public Health, Minneapolis, Minnesota, United States

## Abstract

The National Core Indicators Aging and Disability (NCI-AD) survey samples home and community-based service recipients to understand their unmet HCBS needs. Using 2016-2019 NCI-AD data (N=24348), we evaluated the demographic (e.g, race), health (e.g, hearing ability), environmental (e.g., living alone) and payer (e.g., Medicaid) factors associated with reporting an unmet need among HCBS users age ≥65 with and without dementia. We defined an unmet need as reporting wanting more adult day services, caregiver support, delivered meals, homemakers, personal care assistance, or transportation services. We modeled the odds of unmet needs and tested for differences in unmet needs between people with and without dementia. Personal care assistance was the most frequently used service among people living with and without dementia (59% vs. 50%) followed by delivered meals (22% vs. 37%), homemaker services (21% vs. 25%), adult day services (12% vs. 8%), transportation (9% vs. 8%) and caregiver support (9% vs. 5%). Unmet needs among people with and without dementia respectively varied between 5% and 3.2% (adult day services) to 11% and 14% (personal care assistants). Asians compared to Whites without dementia were significantly more likely to report an unmet need for personal care and homemaker support, and less likely to report an unmet need for caregiver support, but there was little association for people with dementia. Overall health and hearing ability also had different associations with unmet needs between people living with and without dementia but the relationships were not consistent in direction.

